# Integrated Care for Older People Screening Tool for Measuring Intrinsic Capacity: Preliminary Findings From ICOPE Pilot in China

**DOI:** 10.3389/fmed.2020.576079

**Published:** 2020-11-30

**Authors:** Lina Ma, Jagadish K. Chhetri, Yaxin Zhang, Pan Liu, Yumeng Chen, Yun Li, Piu Chan

**Affiliations:** ^1^Department of Geriatrics, National Research Center for Geriatric Medicine, Xuanwu Hospital, Capital Medical University, Beijing, China; ^2^Department of Neurology and Neurobiology, Xuanwu Hospital, Capital Medical University, Beijing, China

**Keywords:** intrinsic capacity, frailty, older adults, screening, integrated care

## Abstract

**Objectives:** The World Health Organization (WHO) proposed the Integrated Care for Older People (ICOPE) screening tool to identify older people with priority conditions associated with declines in intrinsic capacity (IC). We aimed to determine the clinical utility of the WHO ICOPE screening tool in a Chinese population.

**Method:** A total of 376 adults aged 68.65 ± 11.41 years participated in the study. IC was assessed with the WHO ICOPE screening tool, covering five domains: cognitive, locomotor, sensory, vision, and psychological capacity. We assessed the activities of daily living (ADL); instrumental activities of daily living (IADL); the Fried frailty phenotype; FRAIL scale; Strength, Assistance With Walking, Rising From chair, Climbing Stairs, and Falls (SARC-F) scale; Mini-mental State Examination (MMSE); Geriatric Depression Scale (GDS); social frailty; and quality of life.

**Results:** There were 260 (69.1%) participants who showed declines in one or more IC dimensions. The percentages of decline in mobility, cognition, vitality, hearing, vision, and psychological capacity were 25.3, 46.8, 16.2, 15.4, 11.7, and 12.0%, respectively. IC decreased with increasing age. After adjusting for age, sex, and multimorbidity, participants with declines in IC were more likely to be older, frail, and disabled. They also had worse physical, mental, and overall health. There was a higher prevalence of declines in IC in participants with frailty. After adjusting for age, IC was positively correlated with walking speed, resilience score, and MMSE score and negatively correlated with frailty, SARC-F score, IADL score, GDS score, and physical and mental fatigue. The IC score was not associated with body composition variables such as fat-free mass, body fat percentage, or visceral fat area. Higher IC was associated with better quality of life. The area under the curve of the receiver operating characteristic (AUC-ROC) for the ICOPE screening tool vs. Fried phenotype, FRAIL, ADL disability, IADL disability, and SARC-F were 0.817, 0.843, 0.954, 0.912, and 0.909, respectively.

**Conclusion:** Our research affirms that the ICOPE screening tool is useful to identify adults with poor physical and mental function in a Chinese sample. This tool may assist in identifying declines in IC in an integrative care model and help slow down function decline and onset of care dependence.

## Introduction

The increase in the aging population has emerged as a major global phenomenon. Frailty is an important construct that has been used in geriatric medicine over the past 20 years to conceptualize the age-related increase in vulnerability at an individual level (1, 2). Frailty is defined as a geriatric syndrome characterized by the reduced ability of an individual to maintain physiological homeostasis and increased risks of adverse clinical outcomes (3). Although there is a growing interest regarding frailty related to all geriatric syndromes and diseases, integration of this concept in clinical practice has been impeded due to a lack of unified frailty instruments and variations in research outcomes (4–6).

The disease concept is increasingly being replaced by a functional approach to address the healthcare needs of the older people (7). To overcome the weaknesses of the frailty concept and to better disseminate geriatric care to the aging population, the World Health Organization (WHO) recently proposed a novel model for healthy aging oriented around trajectories of functional ability (8). This model holds great potential to make a significant public health impact, particularly by aiding in healthy aging. The World Report on Aging and Health defines healthy aging as the process of developing and maintaining the functional ability that fosters well-being in old age. Functional ability is proposed to be determined by an individual's environmental factors, physical and mental attributes known as intrinsic capacity (IC) and the interaction between them (9). As the composite of all physical and mental capacities, IC could decline with age due to underlying disease and the aging process. While traditional healthcare models focus on the identification and treatment of medical conditions, IC emphasizes on the positive attributes of individuals, i.e., focuses on maintaining an individual's functional capacities throughout life course rather than treating a particular illness.

IC can be evaluated using five domains: cognition, locomotion, vitality, sensory, and psychological capacities (10). The five domains of IC are related to the reduction in physical and mental capacities and independently predict mortality and care dependence in older adults (10). Thus, it could be intuitive to consider that enhancing IC through life could potentially prevent adverse events and geriatric conditions such as frailty in old age. The WHO Guidelines on Integrated Care for Older People (ICOPE) offers evidence-based recommendations for effective interventions to address declines in physical and mental capacities in older people so as to optimize IC (11). However, questions pertaining to translate theory into practice arise because there is less agreement on how the domains of IC are assessed (12). Very few evidence on the clinical utility of the ICOPE tool is available. International validation studies of the instrument and how to incorporate IC assessment in the new care model are ongoing. It remains unclear how the specific domains, assessed by the new tool, relate to physical and mental functions as well as underlying biological pathways. Thus, in this pilot study, we aimed to determine if the WHO ICOPE screening tool can identify adults with poor physical and mental function in a Chinese population.

## Methods

### Study Design and Participants

This is a cross-sectional study. A total of 376 participants aged 50–97 years admitted to the Department of Geriatrics in Xuanwu Hospital Capital Medical University during 2019 were included. The participants consisted of 224 male and 152 female, with an average age of 68.65 ± 11.41 years. Inclusion criteria consisted of relatively healthy participants without acute illness and aged ≥50 years who were admitted for a physical examination and completed the WHO ICOPE screening assessment. Participants who suffered from acute heart failure, acute infection, acute cerebrovascular disease, severe cardiac, liver or kidney dysfunction, dementia, Parkinson's disease, and mental illnesses or were unable to provide informed consent because of aphasia, deafness, and blindness were excluded. All the participants underwent physical examination and comprehensive geriatric assessment. The clinical and demographic variables were collected using a face-to-face questionnaire by trained staff. All subjects gave written informed consent in accordance with the Declaration of Helsinki, and the study was approved by the ethical review board of Xuanwu Hospital Capital Medical University.

### Measures

#### IC Construct

IC was assessed by the WHO ICOPE screening tool and included the following five domains (11):

1. Cognitive decline was determined if participants provided an incorrect response to either of the two questions on orientation in time and space or if they could not recall the three words they were asked to remember.
2. Limited mobility was defined as being unable to complete five chair rises within 14 s.
3. Malnutrition was defined as weight loss (more than 3 kg over the previous 3 months) or appetite loss.
4. Sensory loss: (a) Visual impairment was defined as any problems experienced with their eyes, difficulties in seeing far, reading, eye diseases, or currently under medical treatment; (b) hearing loss was defined as failing to hear whispers in the whisper test.
5. Depressive symptoms were defined as the participants being bothered by feeling down, feeling depressed or hopeless, or having little interest or pleasure in doing things over the preceding 2 weeks. The impairment of each item was scored as 0 points.

The IC score ranged from 0 to 6, with a higher score representative of better IC, i.e., for the six items indicated above participants were scored either 0 (representing decline) or 1 (representing no decline).

#### Frailty Assessment

Frailty was measured with the frailty phenotype assessment that includes five components: slowness, weakness, exhaustion, weight loss, and low activity (13). Participants who exhibited three or more of these components were classified as frail. The cutoff value for weakness was determined by grip strength after adjusting for sex and body mass index (BMI) (14). Walking speed was measured by a 4-m walk test, and the cutoff value for slowness was determined after adjustment for sex and height (15). Exhaustion was indicated by self-rated responses to questions from the Center for Epidemiological Studies Depression Scale: “I felt that everything I did was an effort” or “I could not get going” (16). Weight loss was defined as a self-reported unintentional weight loss of 3 kg in the past 6 months or as having a BMI <18.5 kg/m^2^. Low activity was defined as self-reported exercise for <3 h/week over the past 12 months (17). The FRAIL scale was used to identify frailty, with individuals scoring three and above being identified as frail (18).

#### Physical Function Assessment

Functional ability was assessed by the Barthel index for activities of daily living (ADL) and instrumental activities of daily living (IADL). An individual's performance on each item was classified as independent, partially dependent, and completely dependent and scored as 1, 2, and 3, respectively. Chronic diseases were defined as self-reported history of chronic disease diagnosed by a doctor. Charlson Comorbidity Index (CCI) was calculated (19). Polypharmacy was defined as taking more than five drugs. The Strength, Assistance with walking, Rising from chair, Climbing stairs, and Falls (SARC-F) questionnaire was used to assess sarcopenia, with higher scores indicating more severe sarcopenia (20).

#### Psychological Function Assessment

Depression was assessed with the 30-item Geriatric Depression Scale (GDS) (21). Cognitive function was evaluated using the Mini-mental State Examination (MMSE) (22). The 25-item Resilience Scale (RS) was used to assess psychological resilience (23), with higher scores reflecting greater resilience.

#### Social Function Assessment

Social frailty (SF) was assessed by the HALFT scale, which was validated in the Beijing Longitudinal Study of Aging and consists of the following five items: unhelpful to others, limited social participation, loneliness, financial difficulty, and not having anyone to talk to. The total score ranges from 0 to 5 points: a score of 0 was considered no SF or robust social function, 1–2 was considered pre-SF, and a score of ≥3 indicated SF (24).

#### Quality of Life

The health-related quality of life questionnaire 5-level EQ-5D (EQ-5D-5L) and the EQ visual analog scale (EQ-VAS) were used to measure generic health status; a higher EQ-VAS score indicates better health status (25). The Pittsburgh Fatigability Scale was used to measure physical and mental fatigue (26).

#### Organ Function Assessment

Heart rate was measured and recorded once every 20 min for 24 h using the active electrocardiography and disassembled by professionals. Peak expiratory flow (PEF) was determined using a spirometer. Bones mineral density (BMD) was determined using a dual-energy X-ray absorptiometry (DEXA) scan (LUNAR iDXA, USA). A single experienced technologist performed all the DEXA scans. BMD was measured at the lumbar spine vertebra L2–L4, femoral neck, and total hip. Bioelectrical impedance analysis, to estimate body composition, was carried out with the ioi353 Composition Analyzer (JAWON, Korea) with 8 points of tactile electrodes according to the manufacturer's guidelines; fat mass, fat-free mass, body fat percentage (BFP), and visceral fat area were obtained.

#### Blood Test

Peripheral blood samples were collected after overnight fasting; serum samples were isolated and were stored at −80°C before use. C-reactive protein (CRP) levels were measured using immunoturbidimetry.

### Statistical Analysis

Data were presented as mean ± standard deviation or mean rank or number and percentage. The difference in the characteristics between the two groups was evaluated using the chi-square test for categorical variables, independent *t*-test for continuous variables, and Kruskal–Wallis comparisons for the abnormally distributed continuous variables. A multivariate logistic regression analysis—adjusted for age, sex, and multimorbidity—was used to identify the factors associated with lower IC. Spearman correlation coefficients were calculated to assess the relationship between IC and the other factors. The area under the curve of the receiver operating characteristic (AUC-ROC) was calculated. All statistical analyses were performed with SPSS (Chicago, IL, USA, version 20.0) or GraphPad Prism 7.0 software (GraphPad Software Inc., CA, USA). *p*-value was considered significant at <0.05.

## Results

**Figure 1:**
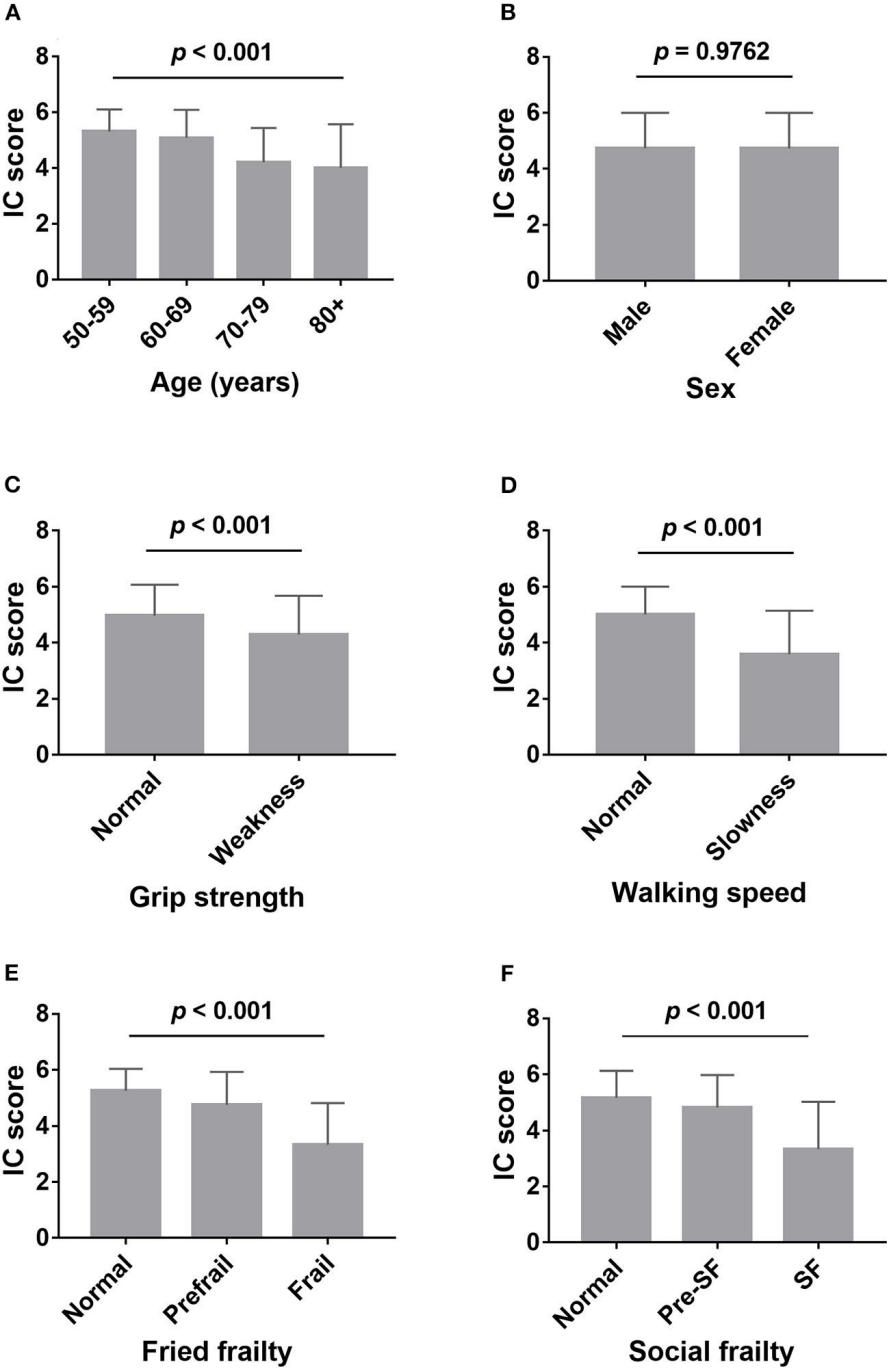
Comparison of intrinsic capacity score between different groups. **(A)** Comparison of intrinsic capacity score among the different age groups; **(B)** comparison of intrinsic capacity score between male and female; **(C)** comparison of intrinsic capacity score between weakness (low grips strength) and normal grip strength; **(D)** comparison of intrinsic capacity score between slowness (slow walking speed) and normal walking speed; **(E)** comparison of intrinsic capacity score among robust, prefrailty, and frailty, assessed by Fried phenotype; **(F)** comparison of intrinsic capacity score among normal, presocial frailty, and social frailty, assessed by HALFT scale. IC, intrinsic capacity; SF, social frailty.

Of the 376 participants, the average IC score was 4.73 ± 1.27, 116 participants (30.9%) were categorized as having no decline in IC. Of the participants, 137 (36.4%), 64 (17.0%), 35 (9.3%), 14 (3.7%), 7 (1.9%), and 3 (0.8%) showed decline in one, two, three, four, five, and six domains, respectively. The percentage of decline in mobility, cognition, vitality, hearing, vision, and psychological domains were 25.3, 46.8, 16.2, 15.4, 11.7, and 12.0%, respectively. Participants' IC score decreased with increasing age, from 5.32 ± 0.79 at age 50–59 to 4.01 ± 1.56 at age 80 and older (Figure 1A). Slow walking speed and low grip strength were associated with worse IC (Figures 1C,D). There was no difference observed in IC between men and women (Figure 1B). Those with physical frailty and social frailty had decreased IC when compared to the nonfrail participants (Figures 1E,F). After adjusting for age, these associations remained unchanged, except for grip strength (*p* = 0.128).

As shown in Table 1, participants with declines in IC were older, had more chronic diseases, and had worse general health as indicated by the EQ-VAS. Participants with declines in IC had worse physical function as indicated by lower Barthel index, walk speed, grip strength, and physical fatigue. They had worse mental function indicated by lower MMSE scores, had higher GDS scores, more mental fatigue, and had worse social function as indicated by higher SF score. There was a higher prevalence of declines in IC in participants with polypharmacy and frailty. However, IC was not affected by sex, CCI, and resilience score. After adjusting for age, sex, and multimorbidity, a decline in IC was associated with declining physical function (Fried frailty, FRAIL score, IADL score, and physical fatigue), mental health (MMSE and GDS), and quality of life (EQ-VAS).

**Table 1:** Characteristics of the DIC and non-DIC participants.

	**Non-DIC (*n* = 116)**	**DIC (*n* = 260)**	***x*2/t/z**	***p*-value**	Adjusted *p*-value^*^
Age (years)	62.47 ± 9.40	71.41 ± 11.16	−8.026	<0.001	–
Gender (*n*, %)					
Male	71 (31.7)	153 (68.3)	0.186	0.667	
Female	45 (29.6)	107 (70.4)			
Number of chronic diseases	4.03 ± 2.91	4.97 ± 2.96	−2.815	0.005	–
CCI	3 (1,4)	3 (2,4)	1.464	0.226	–
EQ-VAS	74.81 ± 12.27	68.63 ± 16.17	6.177	<0.001	0.031
Barthel index	98.30 ± 3.53	91.24 ± 15.39	4.474	<0.001	0.735
IADL score	7.04 ± 0.30	8.34 ± 2.95	−6.992	<0.001	0.039
Walking speed (m/s)	1.00 (0.85, 1.14)	0.92 (0.72, 1.08)	8.298	0.003	0.995
Grip strength (kg)	31.0 (25.2, 40.7)	25.1 (19.9, 35.3)	13.392	<0.001	0.531
MMSE	29.23 ± 1.31	27.22 ± 2.65	9.700	<0.001	<0.001
GDS	2 (1,4)	4 (2,7)	18.898	<0.001	<0.001
Resilience	140.21 ± 15.82	136.83 ± 20.86	1.623	0.106	–
Polypharmacy (*n*, %)					
Yes	38 (20.2)	150 (79.8)			
No	78 (41.5)	110 (58.5)	19.947	<0.001	0.173
Fried frailty (*n*, %)					
Robust	59 (43.4)	77 (56.6)			
Prefrail	55 (29.7)	130 (70.3)	36.168	<0.001	0.014
Frail	2 (3.6)	53 (96.4)			
Social frailty score	0.90 ± 0.88	1.26 ± 1.01	−3.198	0.002	0.120
Physical fatigue	13 (8.5, 23)	23 (14,33)	−5.556	<0.001	0.046
Mental fatigue	11 (0, 21)	14 (1,30)	−2.921	0.004	0.824
FRAIL score	0 (0, 1)	1 (0, 1)	24.428	<0.001	0.018
SARC-F score	0 (0, 0)	0 (0, 2)	10.628	0.001	0.353

*Data were expressed as mean (standard deviation), median (interquartile range) or n (%)*.

* *Adjusted by age, sex, and multimorbidity*.

*DIC, decline in intrinsic capacity; CCI, Charlson Comorbidity Index; EQ-VAS, the EQ visual analog scale; IADL, instrumental activities of daily living; MMSE, the Mini-mental State Examination; GDS, the Geriatric Depression Scale; SARC-F, Strength, Assistance With Walking, Rising From Chair, Climbing Stairs, and Falls*.

Next, we explored whether declines in IC affects organ function. Table 2 shows that participants with declines in IC had higher systolic blood pressure (SBP), higher 24 h heart rate, higher CRP, and lower diastolic blood pressure (DBP). However, no difference was observed after adjusting for age, sex, and multimorbidity. There was no difference in EF, PEF, BMD, and body composition.

**Table 2:** Comparison of organ functions between the DIC and non-DIC participants.

	**Non-DIC (*n* = 116)**	**DIC (*n* = 260)**	***x*2/t/z**	***p*-value**	Adjusted *p*-value^*^
SBP (mmHg)	132.43 ± 15.37	137.44 ± 18.79	−2.719	0.007	0.189
DBP (mmHg)	77.66 ± 11.42	74.86 ± 11.58	2.179	0.030	0.705
BMI (kg/m^2^)	25.55 ± 3.99	25.58 ± 3.69	−0.068	0.946	–
EF (%)	66.24 ± 6.22	65.91 ± 6.87	0.420	0.675	–
24 h HR (/min)	70.53 ± 10.63	66.86 ± 9.37	2.770	0.006	0.071
Fat (kg)	20.08 ± 4.94	20.39 ± 6.25	−0.306	0.760	–
Fat-free mass (kg)	46.51 ± 8.50	46.88 ± 10.50	−0.222	0.825	–
BFP (%)	28.81 ± 5.18	28.40 ± 6.18	0.445	0.657	–
Visceral fat area (cm^2^)	116.08 ± 39.77	124.18 ± 42.82	−1.132	0.259	–
PEF (L/s)	5.46 (4.34,8.11)	5.05 (3.58,6.55)	0.159	0.690	–
BMD (g/cm^2^)	0.86 ± 0.20	0.82 ± 0.17	0.908	0.367	–
CRP (mg/L)	2.30 (1.60,3.40)	2.67 (1.65,4.33)	3.955	0.026	0.239

*Data were expressed as mean (standard deviation), median (interquartile range) or n (%)*.

* *Adjusted by age, sex and multimorbidity*.

*DIC, decline in intrinsic capacity; SBP, systolic blood pressure; DBP, diastolic blood pressure; BMI, body mass index; EF, ejection fraction; HR, heart rate; BFP, body fat percentage; PEF, peak expiratory flow; BMD, bones mineral density; CRP, C-reactive protein*.

**Figure 2:**
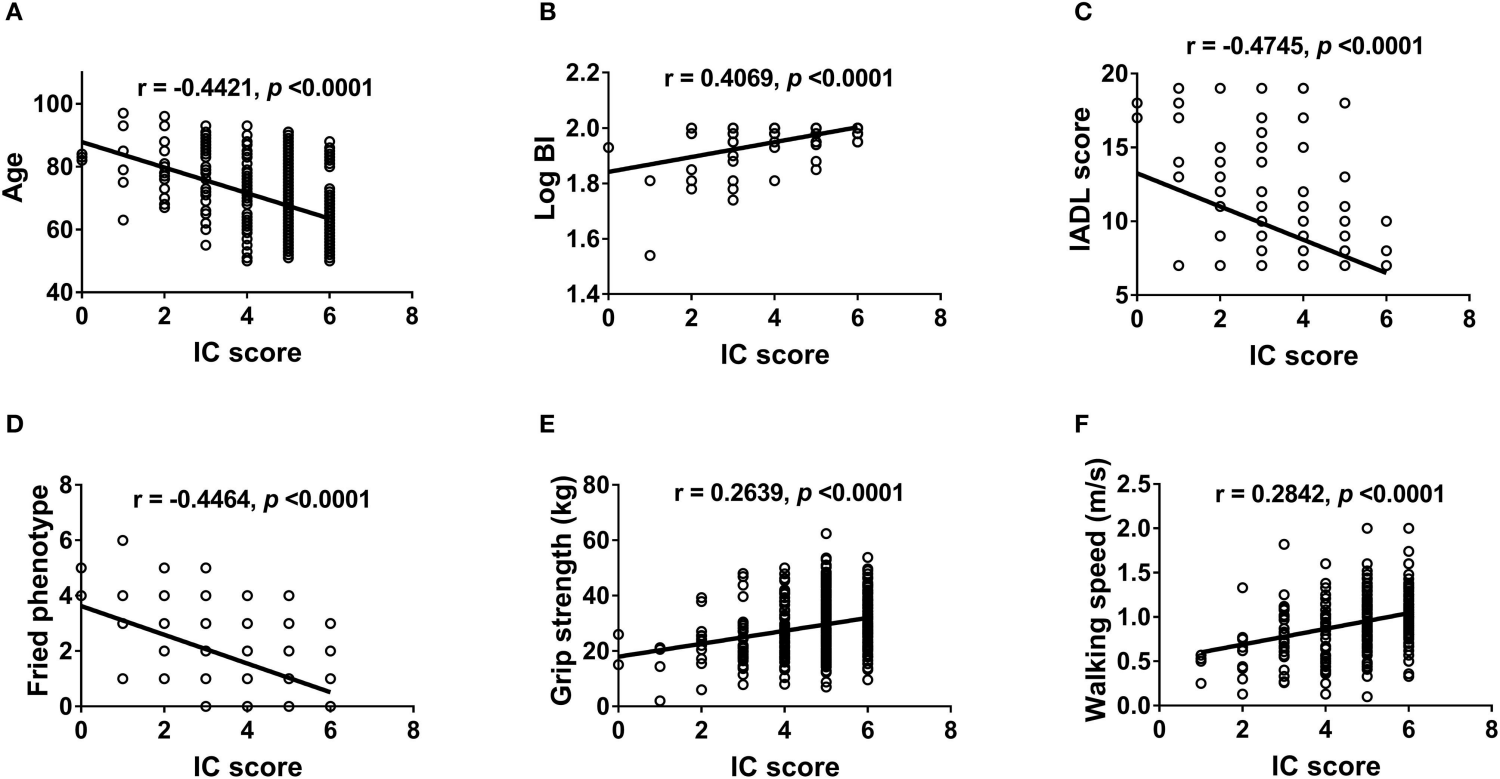
Correlation between intrinsic capacity and physical function. Spearman correlation coefficients were calculated between intrinsic capacity score and **(A)** age, **(B)** Barthel index (log-transformed), **(C)** IADL score, **(D)** Fried phenotype, **(E)** grip strength, and **(F)** walking speed. IC, intrinsic capacity; BI, Barthel index; IADL, instrumental activities of daily living.

**Figure 3:**
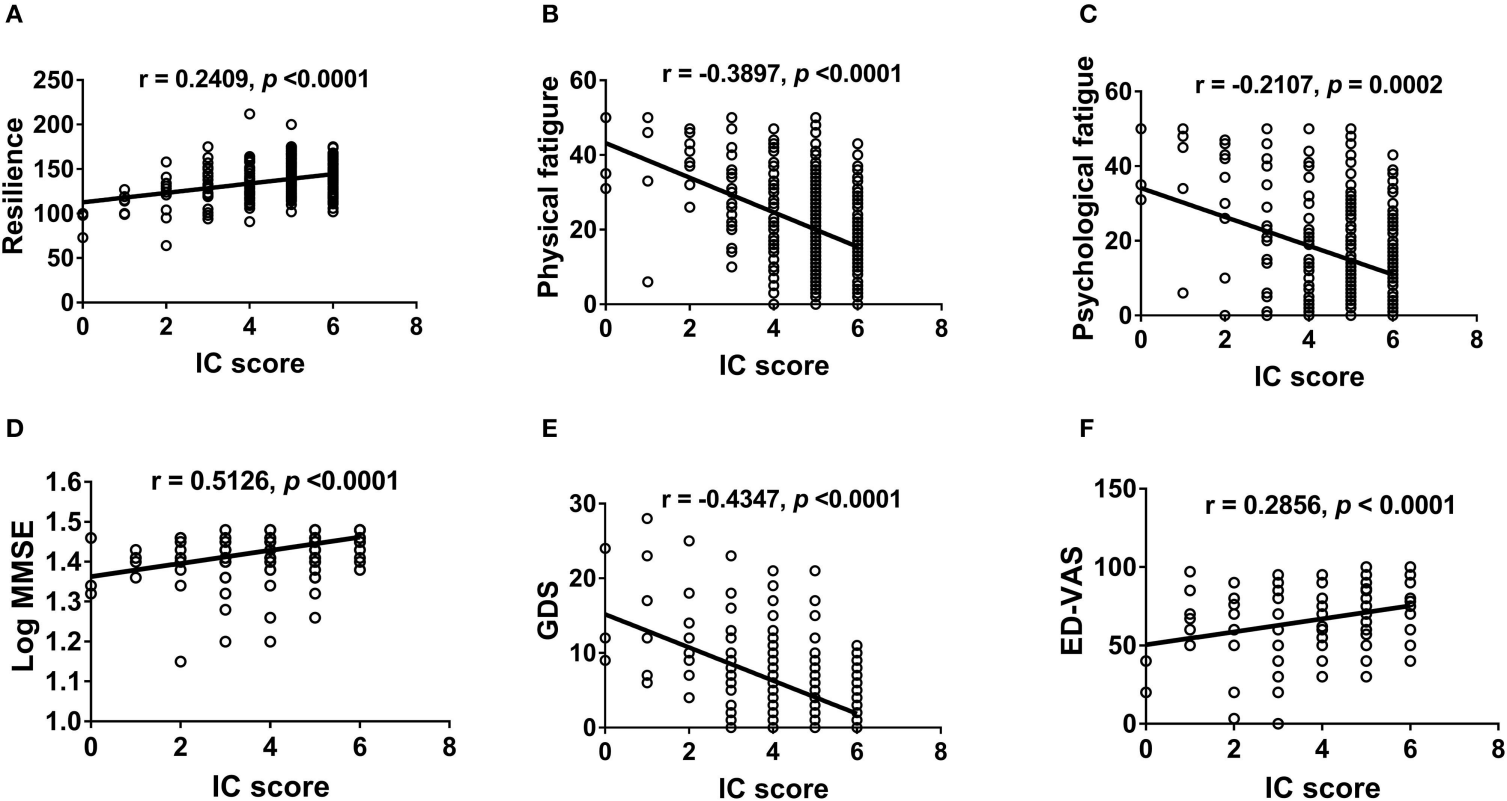
Correlation between intrinsic capacity and psychological health and quality of life. Spearman correlation coefficients were calculated between intrinsic capacity score and **(A)** resilience score, **(B)** physical fatigue, **(C)** mental fatigue, **(D)** MMSE (log-transformed), **(E)** GDS, and **(F)** ED-VAS. IC, intrinsic capacity; MMSE, the Mini-mental State Examination; GDS, the Geriatric Depression Scale; EQ-VAS, the EQ visual analog scale.

**Figure 4:**
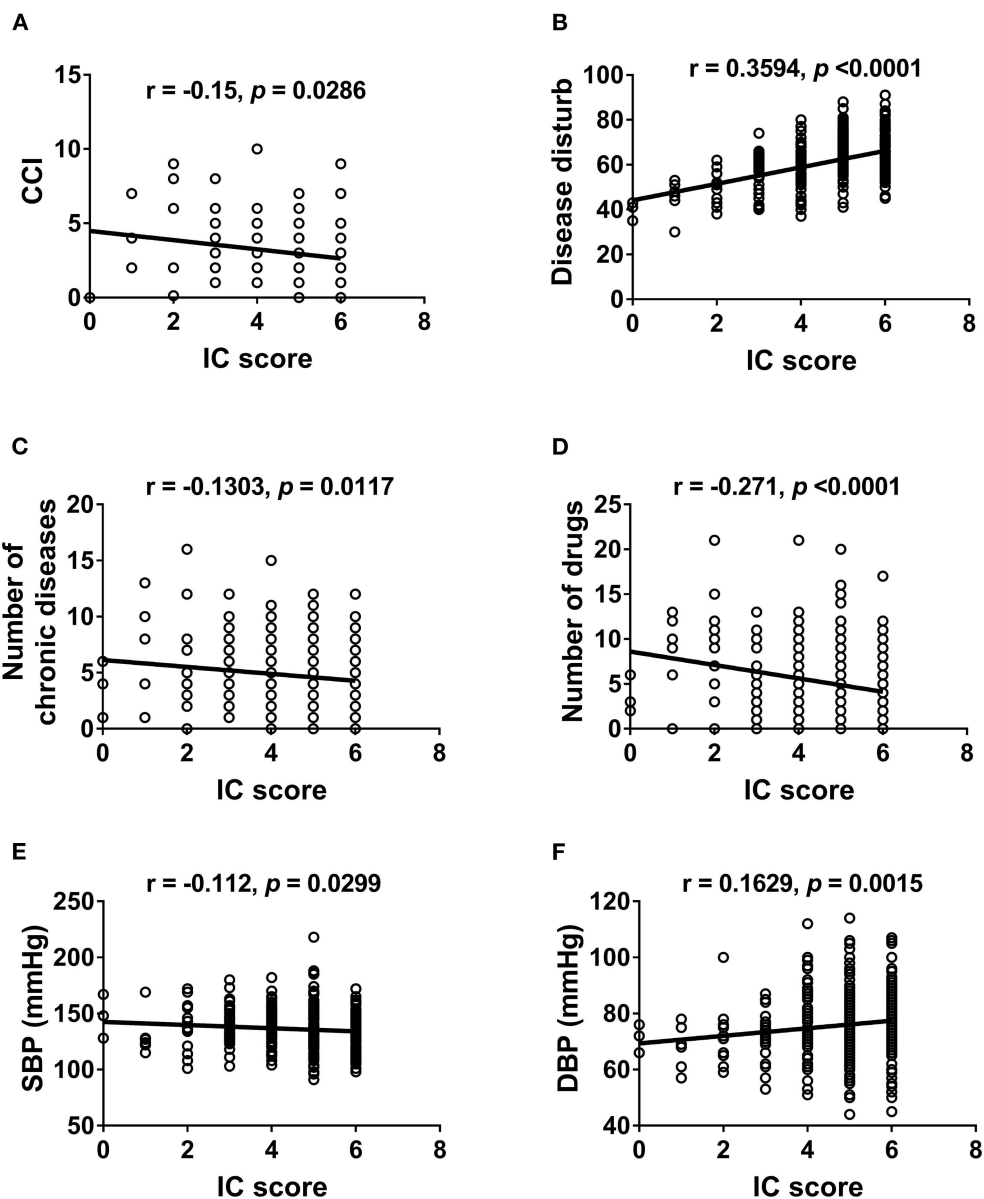
Correlation between intrinsic capacity and chronic diseases. Spearman correlation coefficients were calculated between intrinsic capacity score and **(A)** Charlson Comorbidity Index, **(B)** Disease disturb, **(C)** number of chronic diseases, **(D)** number of drugs, **(E)** systolic blood pressure, **(F)** diastolic blood pressure. IC, intrinsic capacity; CCI, Charlson Comorbidity Index; SBP, systolic blood pressure; DBP, diastolic blood pressure.

We further examined the correlation between IC and physical (Figure 2) and mental functions (Figure 3). IC was significantly positively correlated with the Barthel index, grip strength, walking speed, resilience score, and MMSE score and significantly negatively correlated with age, IADL score, frailty, GDS score, physical fatigue, mental fatigue, and worse quality of life. Further analysis based on chronic diseases showed that the IC score was positively correlated with DBP and negatively correlated with CCI, number of chronic diseases, number of drugs, and SBP (Figure 4). However, after adjusting for age, IC was positively correlated with walking speed (*r* = 0.168, *p* = 0.002), resilience score (*r* = 0.316, *p* < 0.001), and MMSE (*r* = 0.358, *p* < 0.001), while it was negatively correlated with IADL score (*r* = −0.446, *p* < 0.001), Fried frailty score (*r* = −0.398, *p* < 0.001), FRAIL score (*r* = −0.365, *p* < 0.001), SARC-F score (*r* = −0.347, *p* < 0.001), physical fatigue (*r* = −0.278, *p* < 0.001), mental fatigue (*r* = −0.195, *p* = 0.001), and GDS (*r* = −0.552, *p* < 0.001). We further explored the relationship between IC and body composition; however, IC was not associated with fat-free mass, BFP, or visceral fat area.

AUC-ROC was performed to explore the performance of the ICOPE screening tool with respect to frailty, IADL, and ADL. As shown in Table 3, using a cutoff of 4, the sensitivity and specificity of the ICOPE screening tool in identifying frailty defined by Fried phenotype were 90.3 and 50.9%, respectively, and the AUC-ROC for the ICOPE screening tool vs. Fried phenotype was 0.817 (*p* < 0.001). The sensitivity, specificity, and AUC-ROC for the ICOPE screening tool vs. ADL disability were 85.4%, 100%, and 0.954 (*p* < 0.001), respectively, while the sensitivity, specificity, and AUC-ROC for the ICOPE screening tool vs. IADL disability were 90.8%, 73.7%, and 0.912 (*p* < 0.001), respectively.

**Table 3:** The estimating sensitivity, specificity, and AUC-ROC of the ICOPE screening tool.

	**Sensitivity**	**Specificity**	**AUC**	***p*-value**
Frailty (FP)	0.903	0.509	0.817	<0.001
Frailty (FRAIL)	0.875	0.600	0.843	<0.001
ADL disability	0.854	1.000	0.954	<0.001
IADL disability	0.908	0.737	0.912	<0.001
Sarcopenia (SARC-F)	0.867	0.800	0.909	<0.001

*AUC-ROC, under the curve of the receiver operating characteristic; ICOPE, the Integrated Care for Older People; FP, Fried phenotype; ADL, activities of daily living; IADL, instrumental activities of daily living; SARC-F, The Strength, Assistance With Walking, Rising From Chair, Climbing Stairs, and Falls*.

## Discussion

In this pilot study, two-thirds of participants aged 50 years and above experienced one or more declines in IC. IC decreased with increasing age, which is in accordance with the IC model and rationale that an individual's capacities will decline with aging (27). Our results showed that participants with lower IC scores were more likely to be frail, disabled, and have worse quality of life. Frailty due to multiple causes decreases homeostatic reserves and leads to accentuated vulnerability to negative outcomes in older adults (1). The Fried phenotype of frailty considered frailty as an underlying biological syndrome (13), while the frailty index describes frailty as age-related deficits accumulation (28). Interestingly, impairments in some domains in the IC construct are the same as some components of frailty. However, IC and frailty might represent the opposite sides of the same coin. As we have emphasized before, the concept of IC was related to an individual's positive functional status, which is crucial to healthy aging.

This study showed that the IC score was positively correlated with physical function as demonstrated by measures such as walking speed and grip strength, which is consistent with findings from previous research (29). However, IC score was not associated with body composition variables such as fat-free mass, BFP, and visceral fat area; this further indicates that IC may have higher association with muscle function but not muscle mass. Individual's physical decline has been shown to predict mortality and has been considered as an “additional vital sign” in older adults (30). Grip strength predicts decline in physical and mental capacities in older adults. In accordance with our findings, a recent study in Colombia showed that participants with greater handgrip strength had better IC and lower risks of adverse events (29). Moreover, gait speed has a linear relationship with the risk of negative outcomes (31).

Our findings showed that the IC score was negatively correlated with the number of chronic diseases and CCI; moreover, participants with polypharmacy had lower IC. However, after adjusting for age, these factors were no longer statistically different. Previous research has shown that chronic diseases are common in older adults and associated with disability and mortality and that aging increased risk of multimorbidity (32). These factors may have a significant impact on the healthcare burden. However, a lack of geriatric professionals may lead to poor management (under/over treatment) of chronic disease in areas with limited geriatric care resources. Monitoring the trajectories of individuals' physical and mental capacities by collecting preclinical parameters relative to the health profile instead of trying to manage an array of disease diagnoses might overcome such limitations. Data from the English Longitudinal Study on Aging showed that, adjusted for multimorbidity, IC was associated with subsequent functioning (10). The ICOPE approach may enable such a mode of healthcare management when changes in IC suggest the need for clinical intervention.

In our study, IC was positively correlated with MMSE scores and negatively correlated with GDS scores and mental fatigue. Declines in cognitive function are associated with high risks of negative outcomes, and previous studies have shown that cognition can directly affect the other domains of IC. Depressive symptoms have been shown to be strongly associated with functional status (33) and is a risk for subsequent physical decline, even among individuals with no ADL difficulties or mobility disability (34). SF was associated with physical functioning, cognition, and depression and was found to predict mortality in the Beijing Longitudinal Study of Aging (24). This implies that the interactions between mental capacity and social factors may largely contribute to the construct of IC.

Although the present study found that participants with lower IC had higher levels of CRP, the association was lost after adjusting for age. Identifying biomarkers of declines in IC may potentially help develop strategies aimed at preventing the incidence of age-related functional decline and to slow down the progression toward future disability and care dependency. CRP was significantly associated with worse physical function in Colombia, Brazil, and Canada (35), while we previously showed no association between CRP and frailty (36). Data from the Multidomain Alzheimer Preventive Trial showed that baseline low-grade inflammation was related to a 5-year declined IC in older adults (37). In addition, another study found allostatic load to be incrementally associated with IC, even when adjusting for socioeconomic variables and chronic diseases (38). Further studies with large samples on the chronic inflammation markers are needed.

This is the first study in the Chinese population implementing the ICOPE screening tool recommended by the WHO to measure IC. All five domains used in the study were based on the ICOPE guidelines, while in many other studies, the measures included are neither complete nor unified. Furthermore, an inflammation marker was explored since few have operationalized this physiological condition by measuring CRP as we did. We also used the same simple approaches to determine functional deterioration—for example, using the FRAIL and SARC-F scales as validation instruments—and showed that the WHO ICOPE tool is effective in identifying older adults with frailty and sarcopenia. Future studies to determine the utility of the Rapid Geriatric Assessment tool (39) for determining declined IC are warranted. We also showed for the first time the AUC-ROC for the ICOPE screening tool vs. Fried phenotype, FRAIL, ADL disability, IADL disability, and SARC-F to be equally good. However, certain limitations that affect the interpretation of the results should be noted. First, although the participants recruited were relatively healthy and without acute illness, the inpatient population—with an average age of 68.65 ± 11.41 years—may not be representative of the older population in China. Although IC total score was positively correlated with the resilience score, there was no difference in resilience score between individuals with declines in IC and individuals without declines in IC; one potential explanation may be the participants we recruited were relatively healthy. Second, the study is cross-sectional and used a limited sample. Future follow-up studies with a large population should be conducted to validate the WHO ICOPE screening tool as well as explore the longitudinal changes in IC and its relevance to clinical outcomes such as incident disability and other adverse events.

## Conclusion

Early identification of declines in IC is essential to implement interventions that maintain individuals' physical and mental capacities and function or to slow down or reverse the declines in older adults. Our research revealed the WHO ICOPE screening tool can identify those adults with poor physical and mental function in Chinese adults; this indicates that measurement of IC with commonly used instruments is feasible and provides useful information on an individual's physical and mental functioning. By using an integrated care approach, it may be possible to maintain IC and slow down the declines in mobility, nutrition, sensory functions, cognition, and mood, which, in turn, is important to prevent or delay the onset of care dependence. Further studies on the monitoring of IC trajectories and ICOPE approaches are warranted in multiple socioeconomic settings.

## Data Availability Statement

The raw data supporting the conclusions of this article will be made available by the authors, without undue reservation.

## Ethics Statement

The studies involving human participants were reviewed and approved by the Ethical Review Board of Xuanwu Hospital Capital Medical University. The patients/participants provided their written informed consent to participate in this study.

## Author Contributions

LM and PC contributed to the design of the work. LM drafted the manuscript and wrote it together with JC and PC. PL, YZ, YC, and YL recruited the participants. LM, JC, PC, and YL contributed to the analysis and interpretation of data. All authors contributed to writing the paper and revising it critically and gave final approval of this version.

## Conflict of Interest

The authors declare that the research was conducted in the absence of any commercial or financial relationships that could be construed as a potential conflict of interest.

## Abbreviations

ADL: activities of daily living
BFP: body fat percentage
BMD: bones mineral density
BMI: body mass index
CCI: Charlson Comorbidity Index
CRP: C-reactive protein
DBP: diastolic blood pressure
EQ-VAS: the EQ visual analog scale
GDS: Geriatric Depression Scale
IADL: instrumental activities of daily living
IC: intrinsic capacity
ICOPE: Integrated Care for Older people
MMSE: Mini-mental State Examination
PEF: peak expiratory flow
RS: resilience scale
SARC-F: Strength, Assistance With Walking, Rising From Chair, Climbing Stairs and Falls
SBP: systolic blood pressure
SF: social frailty
WHO: World Health Organization.
